# Peripheral CD200R signaling: A critical regulator of post-stroke inflammation in aged mice

**DOI:** 10.1016/j.brainresbull.2025.111686

**Published:** 2025-12-13

**Authors:** Conelius Ngwa, Afzal Misrani, Yan Xu, Jingjing Wang, Rodney Ritzel, Fudong Liu

**Affiliations:** Department of Neurology, The University of Texas Health Science Center at Houston, McGovern Medical School, 6431 Fannin Street, Houston, TX 77030, USA

**Keywords:** Aging, CD200R, CD200, Lymphocytes, Bone marrow chimeras, Ischemic stroke, Neuroinflammation

## Abstract

The immune responses to ischemic stroke are subjected to endogenous inhibitory pathways that delimitate the post-stroke inflammation. Among them, the interaction between CD200 and its receptor (CD200R) is increasingly recognized for its role in regulating neuroinflammation across various central nervous system (CNS) disorders. In the present study, we have examined the role of central (brain) vs. peripheral CD200R signaling in acute ischemic stroke using aged bone marrow chimeric (BMC) mice (16–19 months old). These chimeras were generated by transplanting bone marrow from CD200R knockout (KO), green fluorescent protein (GFP), or wild-type (WT) donor mice into irradiated recipient mice, and then subjected to a 45-min transient middle cerebral artery occlusion (MCAO). At three days post-stroke, flow cytometry, ELISA, and immunohistochemistry (IHC) were used to assess immune responses. Infarct volumes and neurobehavioral deficits were also evaluated. We found that T cell infiltration into the brain was significantly greater in KO-to-GFP (central CD200R signaling) compared to GFP-to-KO (peripheral CD200R signaling) mice. KO-to-GFP mice also exhibited significantly higher levels of pro-inflammatory cytokines IL-1β and TNF-α in the ischemic brain than GFP-to-KO chimeras. Correspondingly, KO-to-GFP mice showed significantly larger brain infarct volumes and worse neurobehavior deficits compared to GFP-to-KO chimeras. Together, these findings indicate that the peripheral (not the central) CD200R signaling plays a critical role in controlling post-stroke immune responses and delineating ischemic injury.

## 1. Introduction

Inflammation is a natural defense mechanism of the body upon pathogenic stimulation, but excessive or prolonged immune activation exacerbates tissue damage and hinders recovery. How to effectively control the immune response in diseases remains a significant challenge. Stroke-induced inflammation is regulated by cell membrane proteins that can lead to increased blood-brain barrier disruption, infiltration of peripheral immune cells, and neuronal damage (Candelario-Jalil, Dijkhuizen, and Magnus, 2022; Iadecola and Anrather, 2011). Cluster of Differentiation-200 (CD200) and its receptor CD200R are membrane-associated glycoproteins that play a significant role in neuroinflammation (Kotwica-Mojzych et al., 2021; Manich et al., 2019; Ngwa and Liu, 2019; Vaine and Soberman, 2014; Zhao et al., 2020). Within the CNS, CD200 is expressed primarily by neurons and endothelial cells (Hernangómez et al., 2014; Morgan et al., 1999; Ngwa and Liu, 2019; Rütsche et al., 2022). CD200 interacts with CD200R expressed by immune cells to exert inhibitory effects on neuroinflammation (Minas and Liversidge, 2006; Ritzel et al., 2019; Zhao, Li, and Sun, 2019). In neurodegenerative disease studies, neuronal CD200 was proposed to bind to CD200R expressed on microglia to quench neuroinflammation (Dentesano et al., 2014; Lehrman et al., 2018; Lyons et al., 2007; Walker, Dalsing-Hernandez, Campbell, and Lue, 2009). However, recent studies found adult microglia express extremely low level of the receptor (Koning, Swaab, Hoek, and Huitinga, 2009; Ngwa and Liu, 2019; Ritzel et al., 2019; Walker and Lue, 2013), and peripheral immune cells express abundant CD200R after stroke (Pujol et al., 2024; Ritzel et al., 2019). This has led to our hypothesis that CD200-CD200R inhibitory axis in peripheral immune cells plays critical role in controlling post-stroke inflammation.

Stroke is a disease that mainly affects the elderly, and how the CD200-CD200R signaling functions in stroke in the aged is not clear. To test the effects of central (brain) or peripheral CD200-CD200R signaling in aged stroke, we have generated three types of aged bone marrow chimeras (16–19 months old), by using bone marrows from CD200R global knockout (KO), wild-type (WT), and Green Fluorescent Protein (GFP)-expressing mice. These chimeras are KO-to-GFP to test central CD200-CD200R signaling, GFP-to-KO for peripheral signaling, and GFP-to-WT as a control chimera. Our findings demonstrate that peripheral CD200-CD200R, rather than central inhibitory signaling, plays a predominant role in regulating immune responses to ischemic stroke in aged mice.

## 2. Materials and methods

### 2.1. Animal models

CD200R1^+ /−^ mice (bred on a C57BL/6 J background) were generously provided by Professor R. Gorczynski (Toronto University, Canada) (Boudakov et al., 2007) and bred as in ( Ritzel et al., 2019), to obtain CD200R1 ^−/−^ mutants (KO). GFP and WT mice were purchased from Jackson Laboratory. All mice were group-housed under pathogen-free conditions with a 12-to-12-h day-night cycle and had access to food and water *ad libitum*. All mice were housed to 15 months old, and then randomly chosen for bone marrow chimera generation (below). All studies were conducted in accordance with NIH guidelines for the care and use of laboratory animals and approved by the Institutional Animal Care and Use Committee (IACUC) of the University of Texas Health Science Center at Houston McGovern Medical School.

### 2.2. Bone marrow chimera (BMC) generation

**Fig. 1.**
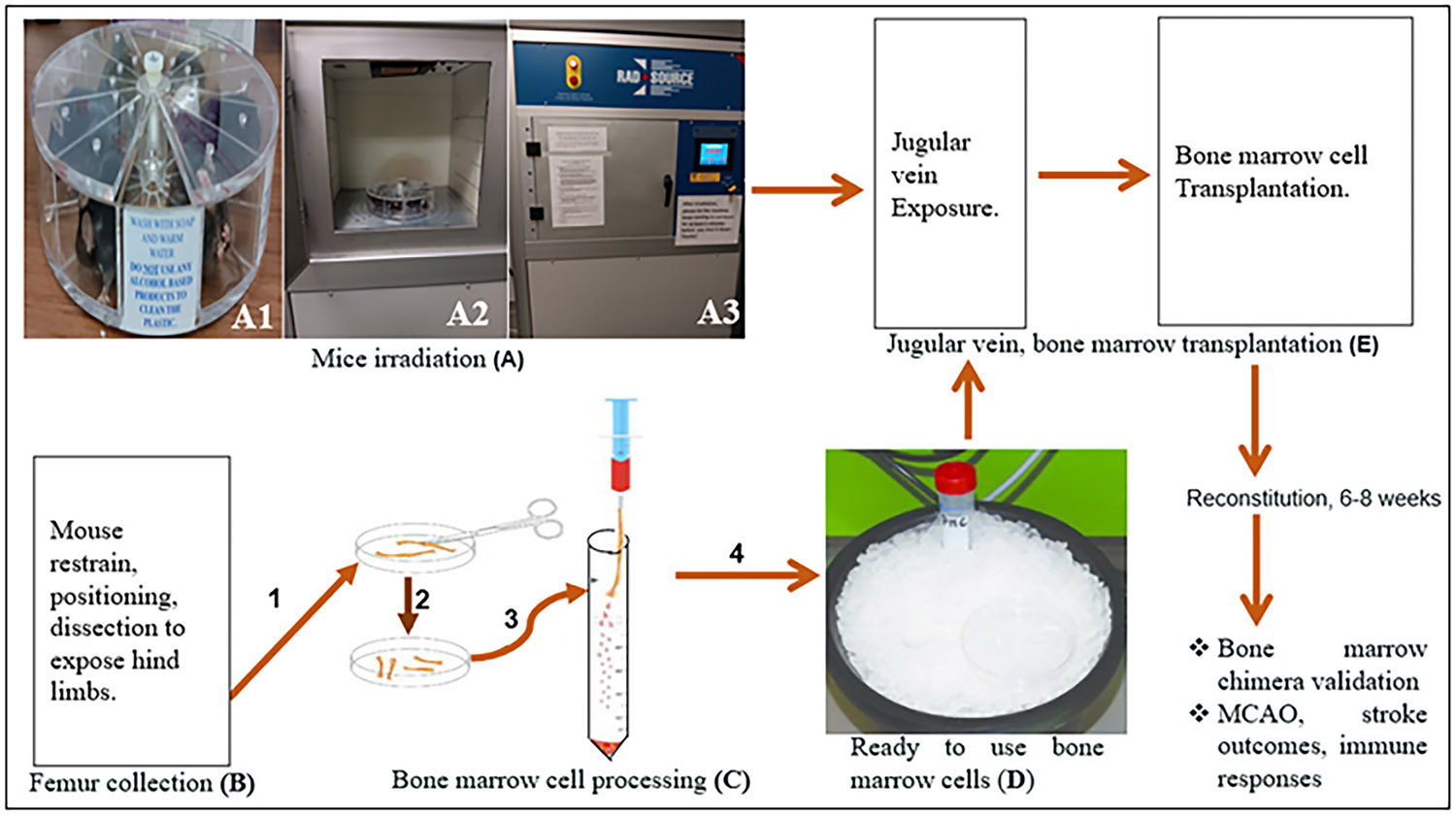
Generation of bone marrow chimeras by X-ray irradiation of mouse, followed by jugular vein transplantation of BM leucocytes. Assemble of mice in Pie Cage and X-ray irradiation (A1-A3), preparation of bone marrow (BM) leucocytes (B-D), transplantation of BM leucocytes into irradiated mouse via jugular vein (E).

Bone marrow chimeras were generated as in (Ngwa et al., 2024; Rodney Ritzel et al., 2018) with modifications. Briefly GFP, C57BL/6 (WT) and KO mice (recipient; 15 months-old) were assembled in a mouse Pie Cage (MCP-1, Braintree Scientific INC). The mice were spaced apart by allowing an empty compartment between each one, ensuring that radiation could reach the entire body of each mouse (Fig. 1,A1). The mice were subjected to X-ray irradiation (Fig. 1,A2-A3), receiving two doses of 250 cGy (~2.3 min each) separated by a 4-hour interval. All animals were exposed to a total dose of 500 cGy to achieve complete myeloablation. After irradiation, the immune cells of the recipient (irradiated) mouse were replaced by transplanting BM cells prepared from femur of donor male or female mouse (Bi et al., 2021; Dos Santos et al., 2010; Ngwa et al., 2024) Rodney (Ritzel et al., 2018). Transplantation of the cells to recipient mice were performed under the microscope through jugular vein injection (Fig. 1 B-E). All experimental mice received prophylactic antibiotics in their drinking water, consisting of sulfamethoxazole and trimethoprim oral suspension, for 2 weeks post-treatment. Chimeras were allowed to reconstitute for 6–8 weeks after the jugular vein injection (JVI). Following BM reconstitution, only the chimeras with > 78 % donor-origin peripheral leukocytes (determined by flow cytometry analysis of these immune cells in blood) were included in the study. The experimental groups consisted of three chimeras: GFP-to-WT, GFP-to-KO, and KO-to-GFP. The non-head-shielded chimeric model allows complete bone-marrow reconstitution, including the skull, while minimizing irradiation-induced injury, blood-brain barrier disruption, and neuroinflammation.

### 2.3. Ischemic stroke model

Cerebral ischemia was induced in mice by reversible middle cerebral artery occlusion (MCAO) and under isoflurane anesthesia as previously described (Al Mamun et al., 2020; Liu, Schafer, and McCullough, 2009; Qi et al., 2023). Briefly, a midline ventral neck incision was made, and unilateral MCAO was performed by inserting a 6–0 silicone-coated suture into the right internal carotid artery 6 mm from the internal carotid/ pterygopalatine artery bifurcation via an external carotid artery stump. Reperfusion was performed by withdrawing the suture 45-min after the occlusion. Rectal temperature was maintained at 36.5 ± 0.5 °C during surgery with an automated TC-1000 temperature-control feedback system (CWE, Inc., Ardmore, PA, USA). All mice were monitored on a daily basis and then sacrificed at 3 days post-stroke, for analysis of stroke outcomes and immune responses. Sham-operated animals underwent the same procedure including exposure to isoflurane and a midline ventral neck incision, but the suture was not advanced into MCA. Laser Doppler flowmetry (Moor Instruments Ltd, UK) was applied to measure CBF through the skull at the right temporal fossa. Only the mice whose CBF showed a drop of over 85 % of baseline after MCAO was included in the following experiments. The mortality after MCAO was 35 % after 3 days stroke. The size of the MCAO-induced infarct was measured by Cresyl violet (CV) staining as described in (Qi et al., 2023).

### 2.4. Flow cytometry (FC)

Flow cytometry was performed as previously described with modifications (Ngwa et al., 2022). Briefly mice were euthanized and transcranial perfused with 1 % heparin in cold PBS, and the brains were harvested. The ipsilateral hemispheres were diced and placed in complete RPMI 1640 (cat # 30–200, ATCC) medium and mechanically and enzymatically digested in collagenase/dispase (1 mg/mL) and DNAse (10 mg/mL), for 1 h and at 37 °C. The cell suspension was diluted in regular RPMI 1640 and then filtered through a 70 μm filter and placed into a 70 %/ 30 % Percoll gradient. Cells were harvested from the interphase portion of the gradient, washed in PBS (1x), and blocked with purified rat anti-mouse CD16/CD32 (mouse BD FC block, cat # 553142) in ice. The blocked cells were stained using primary antibody-conjugated fluorophores including: anti-CD11b PE-Cy7 (cat # 101216, BioLgend), Anti-CD45 eF450 (cat # 48–0451–82, ThermoFisher), anti-Ly6C PerCP-Cy5.5 (cat # 128012, BioLegend), anti-Ly6G PE-eFluor 610 (cat # 61–9668–82, ThermoFisher), anti-CD 19 AF 700 (cat # 56–0193–82, ThermoFisher), and anti-CD3 Brilliant Violet 605 (cat # 100237, BioLegend). For BMC validation in blood samples, we followed the same procedure as in (Ritzel et al., 2019), with CD45, CD11b, and GFP channels. For live/dead cell discrimination, LIVE/-DEAD^™^ Fixable Aqua Dead Cell Stain Kit, for 405 nm excitation (cat # L34957, ThermoFisher), was used. Fluorescence minus ones (FMOs) and beads compensations were used for all staining experiments. Data were acquired on Cytoflex_AS41045 (Beckman Coulter) and analyzed using FlowJo (Treestar Inc.).

### 2.5. Enzyme-linked Immunosorbent assay (ELISA)

We used the same procedure as in (Ngwa et al., 2022; Qi et al., 2021) with modification. Briefly blood samples were obtained by cardiac puncture with EDTA-soaked syringed-needles and then centrifuged at 15000 RPM for 20 min, and at 4 °C. Brain tissue in non-pyrogenic 5 mL polystyrene round-bottom tubes (Ref # 352235, Corning USA) were homogenized using glass pistons, in complete NP40 buffer, and also centrifuged at 15000 RPM for 20 mins, and at 4 °C. After centrifugation, the supernatant was collected and analyzed with Nunc^™^ MaxiSorp^™^ ELISA plates_423501 and the ELISA MAX^™^ Deluxe kits. Included in the kits were TNF_430904, IL-1β_432604, IL-4_431104 and IL-10_431414 (BioLegend USA). For brain cytokine assays we used 5 μg total protein in 100 μL final volume, quantified by Bicinchoninic Acid (BCA) protein assay. Signals were measured at 450 nm in EnSpire^™^ Multimode Plate Reader (Perkin Elmer USA).

### 2.6. Neurologic deficit scores (NDS)

Neurological deficits were assessed by the Benderson score system from 0 to 4 as in (Al Mamun et al., 2020; Ngwa et al., 2022). Briefly 0-no deficit; 1-forelimb weakness, torso turning to the ipsilateral side when held by the tail; 2-circling to the affected side; 3-unable to bear weight on affected side, and 4-no spontaneous activity or barrel rolling.

### 2.7. Open field

The open field test (OFT) is a common measure of exploratory behavior, general activity and anxiety-like behavior in rodents, where both the quality and quantity of the activity can be measured (Kraeuter, Guest, and Sarnyai, 2019). Briefly, mice were placed in a single arena facing the middle of a wall. Mice were allowed to explore the arena for 20 min (Qi et al., 2023). After the 20 min duration, the mice were returned to the home cage and arena cleaned with 70 % ethanol. The distance moved was analyzed as the locomotor and exploratory behavior of the mice.

### 2.8. Grip strength

We used the conventional forelimb grip strength test to assess motor function in the mice (Cabe, Tilson, Mitchell, and Dennis, 1978; Meyer, Tilson, Byrd, and Riley, 1979; Smith, Hicks, Ortiz, Martinez, and Mandler, 1995). Briefly, a mouse was gently pulled by its tail ensuring the mouse grips the top portion of the grid and the torso remains horizontal and record the maximal grip strength value of the mouse that is displayed on the screen. This procedure was repeated 3 times to obtain 3 forelimb grip strength measurements for each mouse, and the average strength was calculated.

### 2.9. Immunohistochemistry (IHC)

Immunohistochemical staining was performed for T cell subsets in the brain after three days stroke, as previously described (Qi et al., 2023) with minor modifications. Briefly, mice brains were removed after perfusion, post-fixed in 4 % PFA for 24 h, and cut using a freezing microtome. The brain slices were blocked in 0.3 % Triton X-100, 1 % bovine serum albumin (BSA), and 5 % donkey serum for 2 h at room temperature. The tissue was then incubated overnight at 4°C with the primary antibodies: mouse anti-CD3 (cat# 14–0038–82, 1:100; Thermo Fisher Scientific), rat anti-CD8α (cat# 74–0029 T, 1:200; Thermo Fisher Scientific), and rabbit anti-FoxP3 (cat# 12653, 1:400; Cell Signaling Technology). Secondary antibodies include: donkey anti-mouse Alexa Fluor 594, donkey anti-rat Alexa Fluor 488, and donkey anti-rabbit Alexa Fluor 647 (1:400; Thermo Fisher Scientific). Nuclei were counterstained with DAPI (Thermo Fisher Scientific). Fluorescence images were acquired from three random 20 × fields per animal, and within the peri-infarct area (inner boundary zone of the infarct). Quantitative analysis of fluorescence intensity was performed by an unbiased, blinded investigator using ImageJ software (NIH, version 1.52a). Values were normalized to background signal.

### 2.10. Statistical analysis

Data from individual experiments were presented as mean ± SD, and assessed by Student’s t test, One-way ANOVA or two-way ANOVA with Tukey post hoc test for multiple comparisons using GraphPad Prism Software 10.1.2 (324). P < 0.05 was considered statistically significant. Investigators were blinded to mouse strains for stroke surgery, behavioral testing, infarct, and inflammation analysis.

## 3. Results

### 3.1. Validation of BMC mouse model

**Fig. 2.**
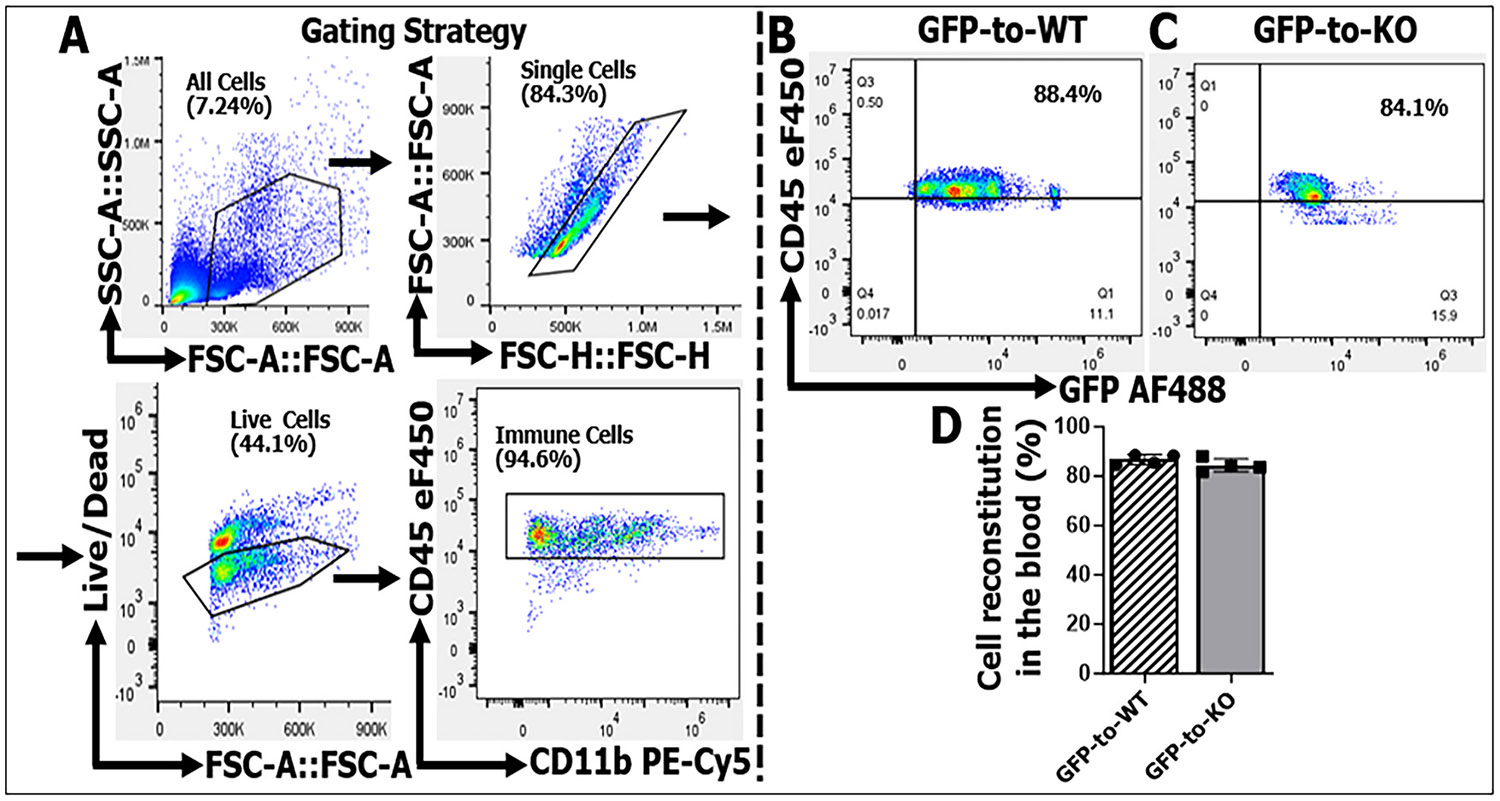
Validation of bone marrow chimerism in WT and CD200R KO recipient mice with transplantation of GFP^+^ donor cells. A, gating strategy for live immune cells in peripheral blood, defined as (CD45^+^CD11b^+^). B,C; representative flow cytometry plots showing the proportion of GFP^+^ donor-derived immune cells among total CD45^+^ cells in GFP donor-to-WT recipient (B) and GFP donor-to-KO recipient (C). D, quantification of GFP^+^ cell reconstitution in peripheral blood, showing >80 % of GFP^+^ leucocytes in both WT and KO recipients. n = 4 mice per group. GFP-to-WT, peripheral and central CD200R intact; GFP-to-KO, peripheral CD200R intact and central CD200R absent.

We generated three different BMC mouse models for this study by transplanting ≥ 1 × 10^6^ immune cells from donor to recipient mouse as in (Ngwa et al., 2024): (i) GFP-to-KO chimeric mice were produced by transplanting BM from mice expressing GFP to irradiated CD200R KO mice. This leads to CD200R deleted in brain immune cells but intact in the peripheral leukocytes (examining the effect of peripheral CD200R signaling). (ii) KO-to-GFP chimeras were produced by transplanting BM from CD200R KO mice to irradiated GFP mice. This chimera lacks CD200R expression in peripheral leukocytes but retains intact CD200R in the brain (central CD200R signaling). (iii) GFP-to-WT mice were produced by transplanting BM from GFP mice to irradiated WT mice. This chimera has intact CD200R throughout the body and serves as a control for the forementioned two chimera types. We performed flow cytometry (FC) on blood samples of these chimeras to validate the BM reconstitution efficiency. The gating strategy of FC is shown in Fig. 2 A. The FC result showed > 80 % GFP-positive peripheral leukocytes in the blood from GFP-to-WT and GFP-to-KO (Fig. 2 B-D), indicating successful BM reconstitution in the chimeras. Similar validation data were seen in brain infiltrating immune cells (ratio of GFP^+^ cell number over total infiltrating immune cell number) (Suppl. Fig. 1).

### 3.2. CD200R deficiency in peripheral immune cells increases T cell infiltration into the brain after stroke

**Fig. 3.**
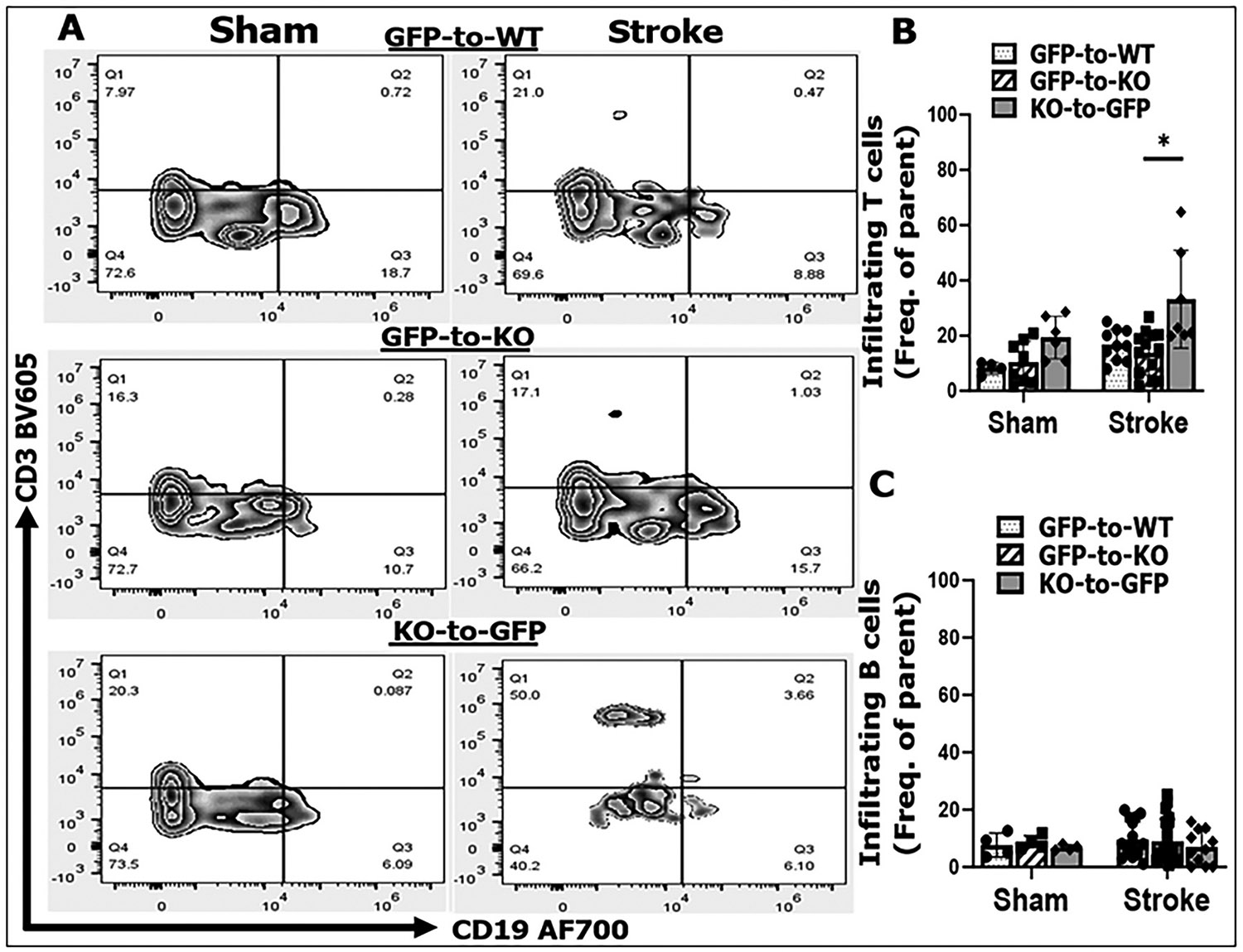
Leucocyte infiltration in ischemic brains of chimeras after 3-days MCAO by flow cytometry. A, representative flow plots of CD3/CD19-gated lymphocytes in sham and stroke mouse brains.B&C, quantification of T cell(B)and B cell (C) frequency of parent. n = 4–6 for sham and 10–14 for the stroke group. Ordinary two-way ANOVA (DF = 1, F (1, 42) = 8.722, p = 0.0051. Sidak’s multiple comparisons test (KO-to-GFP vs. GFP-to-KO),*t* = 4.436, DF = 42, adjusted *p = 0.001. GFP-to-WT, peripheral and central CD200R intact; GFP-to-KO, peripheral CD200R intact and central CD200R absent; KO-to-GFP, peripheral CD200R absent and central CD200R intact.

After stroke, a significant number of peripheral immune cells infiltrate into the brain To investigate whether the absence of CD200R in peripheral immune cells facilitates the immune cell infiltration, we performed MCAO in all three types of BMCs (GFP-to-KO, KO-to-GFP, and GFP-to-WT) and FC was performed on brain samples 3 days after stroke. A significant increase in T cell infiltration was observed in KO-to-GFP compared to GFP-to-KO chimeras, a pattern that was absent in B cells (Fig. 3A-C). Surprisingly monocyte infiltration was not increased in KO-to-GFP vs. GFP-to-KO or GFP-to-WT mice (Suppl. Fig. 2), suggesting monocyte activation may be subjected to multiple regulatory signaling pathways. These findings indicate that deletion of CD200R in peripheral immune cells specifically enhances T cell infiltration into the brain after stroke.

### 3.3. CD200R-deficient peripheral immune cells exacerbate pro-inflammatory responses in the brain after stroke

**Fig. 4.**
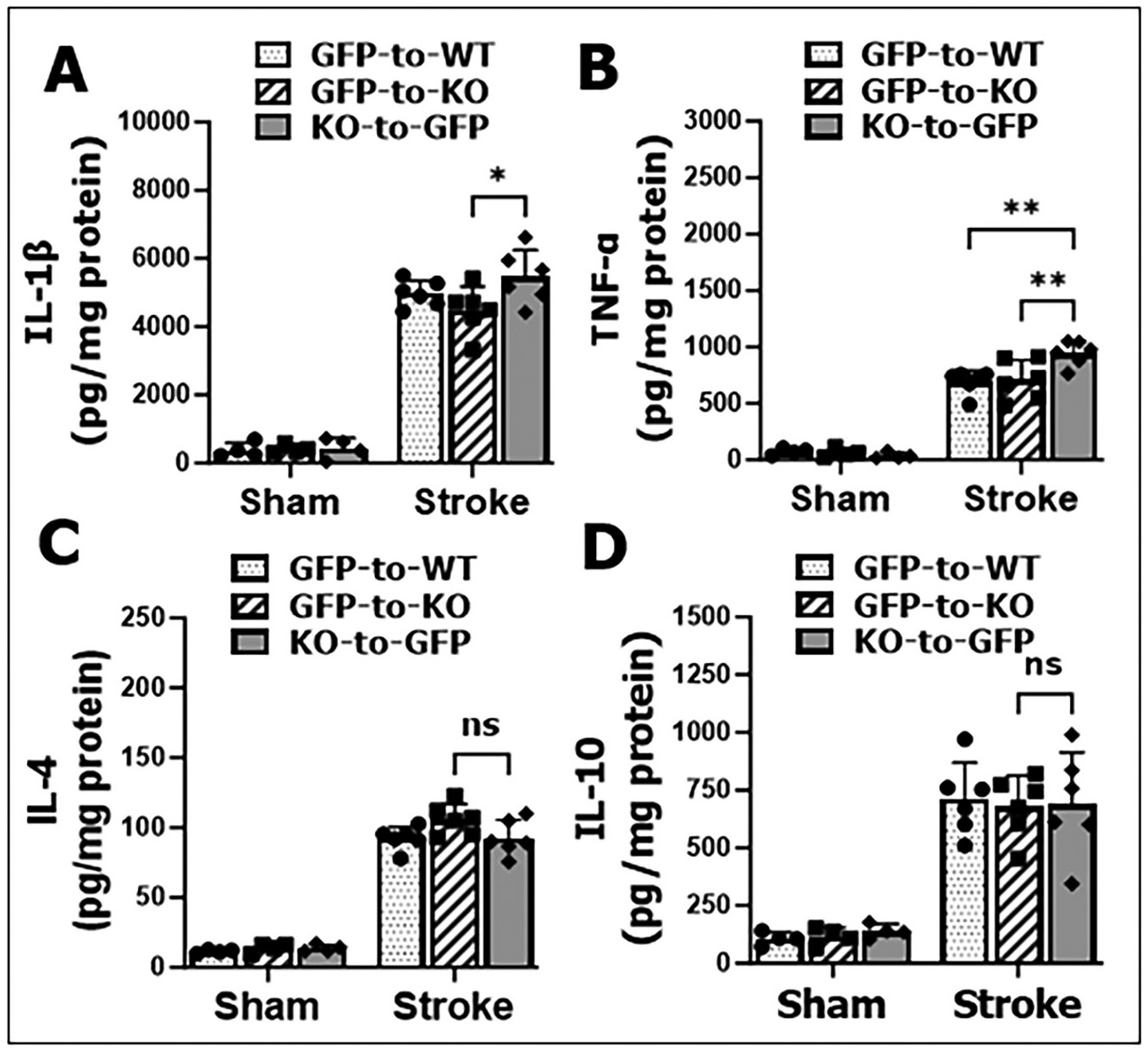
Brain cytokine levels after ischemic injury in BMC mice. A, B; proinflammatory (IL-1β and TNF-α) and C, D; anti-inflammatory (IL-4 and IL-10) cytokine levels were measured in the brain lysates of sham/stroke BMC mice at 3-day poststroke. n = 4 sham and 6 stroke animals per group. Ordinary two-way ANOVA (*IL-1β*: DF = 1, F (1, 24) = 540.7, p < 0.0001; *TNF-α*: DF = 1, F (1, 24) = 325.8, p < 0.0001).

Stroke injury induces inflammatory responses in the ischemic brain (Anrather and Iadecola, 2016; Kawabori and Yenari, 2015; Simats and Liesz, 2022). To assess this response in the KO-to-GFP (central signaling) compared to GFP-to-KO (peripheral signaling) mice, we measured cytokine levels in brain lysates from the ipsilateral hemispheres after 3 days of stroke. Levels of pro-inflammatory cytokines IL-1β and TNF-α were significantly elevated in KO-to-GFP mice compared to GFP-to-KO mice. Interestingly, TNF-α level was also significantly higher in KO-to-GFP (central CD200R intact) vs. GFP-to-WT control (central CD200R intact, peripheral CD200R intact) but not between GFP-to-KO vs. controls (central CD200R absent) (Fig. 4 A, B). Levels of anti-inflammatory cytokines IL-4 and IL-10 did not differ significantly between chimeric types (Fig. 4 C, D). These findings suggest that the absence of peripheral CD200R (in KO-to-GFP mice), despite intact central CD200R signaling, drives an exacerbated pro-inflammatory response in the brain after stroke. The pattern of TNF-α levels between KO-to-GFP vs. GFP-to-WT mice was absent in GFP-to-KO vs. controls, indicating that CD200R signaling in peripheral leucocytes is more important than central CD200R signaling in controlling post-stroke inflammation.

### 3.4. CD200R-deficient peripheral immune cells worsen stroke outcomes

**Fig. 5.**
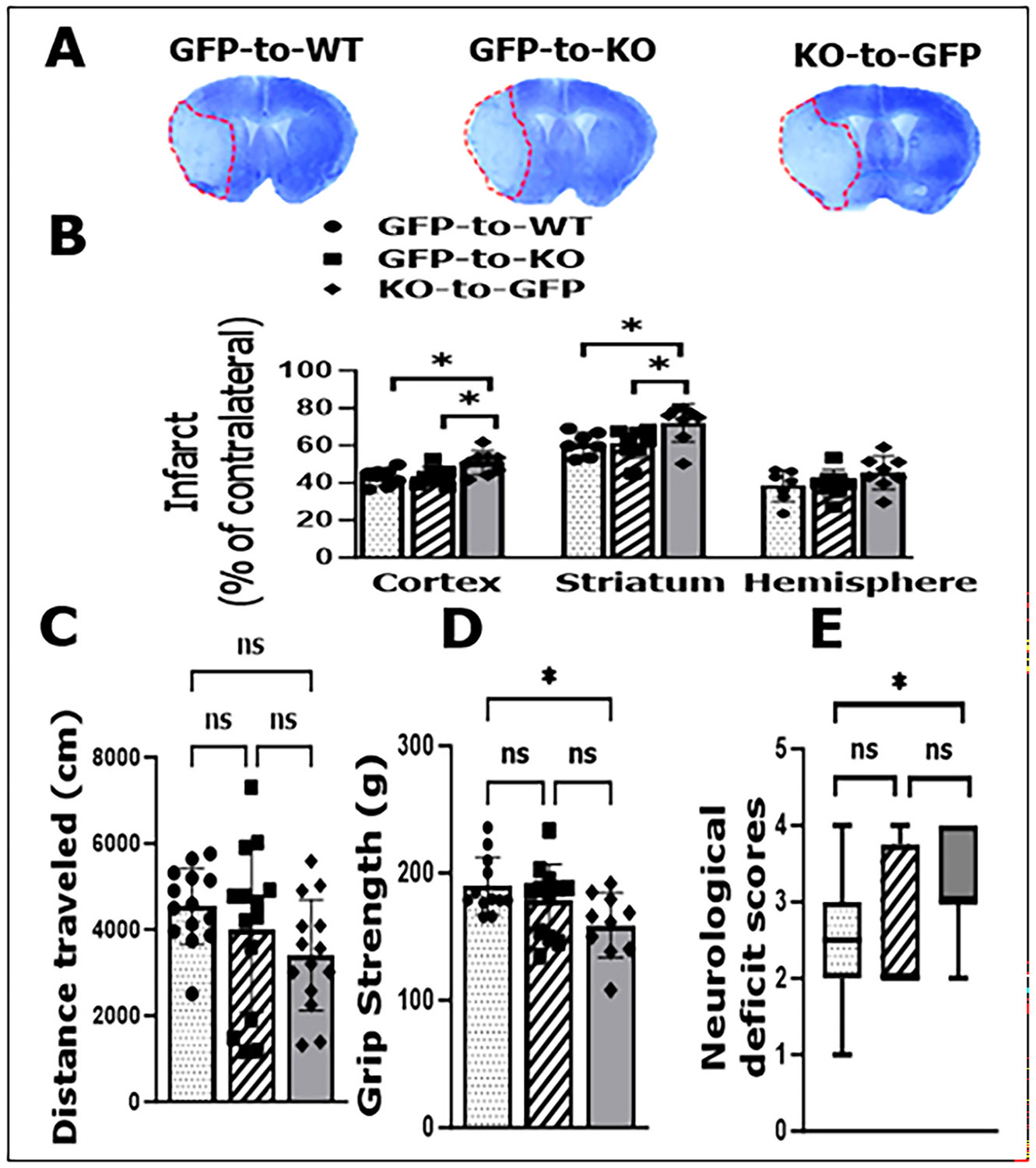
Stroke outcomes in BMC mice after 3-day stroke. A, representative GFP-to-WT, GFP-to-KO, and KO-to-GFP, BMC brain slices stained with Cresyl violet. B, quantification of infarct volumes in cortex, striatum and hemisphere. C, distance traveled in open field test; D, grip strength test; and E, neurological deficits scores. n = 8–14 per group. Ordinary one-way ANOVA (Cortex: F (2, 20) = 4.902, p = 0.0185; Striatum: F (2, 20) = 4.679, p = 0.0215; Grip strength: F (2, 31) = 4.057, p = 0.0272; Neurological deficit scores: F (2, 31) = 3.170, p = 0.0559).

We also examined the effect of peripheral CD200R signaling on stroke outcomes by assessing infarct volumes and behavior tests after stroke. We found significantly larger infarcts in the cortex and striatum, in KO-to-GFP vs. GFP-to-KO or GFP-to-WT chimeras (Fig. 5A, B). KO-to-GFP chimeras also had significantly weaker grip strength and higher NDS scores than GFP-to-WT mice, but no difference was found in the distance traveled in open field test (Fig. 5 C-E). These differences were not seen when GFP-to-KO was compared to GFP-to-WT group. Our data indicate that the deletion of CD200R on peripheral immune cells (but not in brain immune cells) has detrimental effects on stroke outcomes.

### 3.5. CD8α^+^CD3^+^ and FoxP3^+^CD3^+^ T cells exacerbate brain injury, promote neuroinflammation, and worsen stroke outcomes

**Fig. 6.**
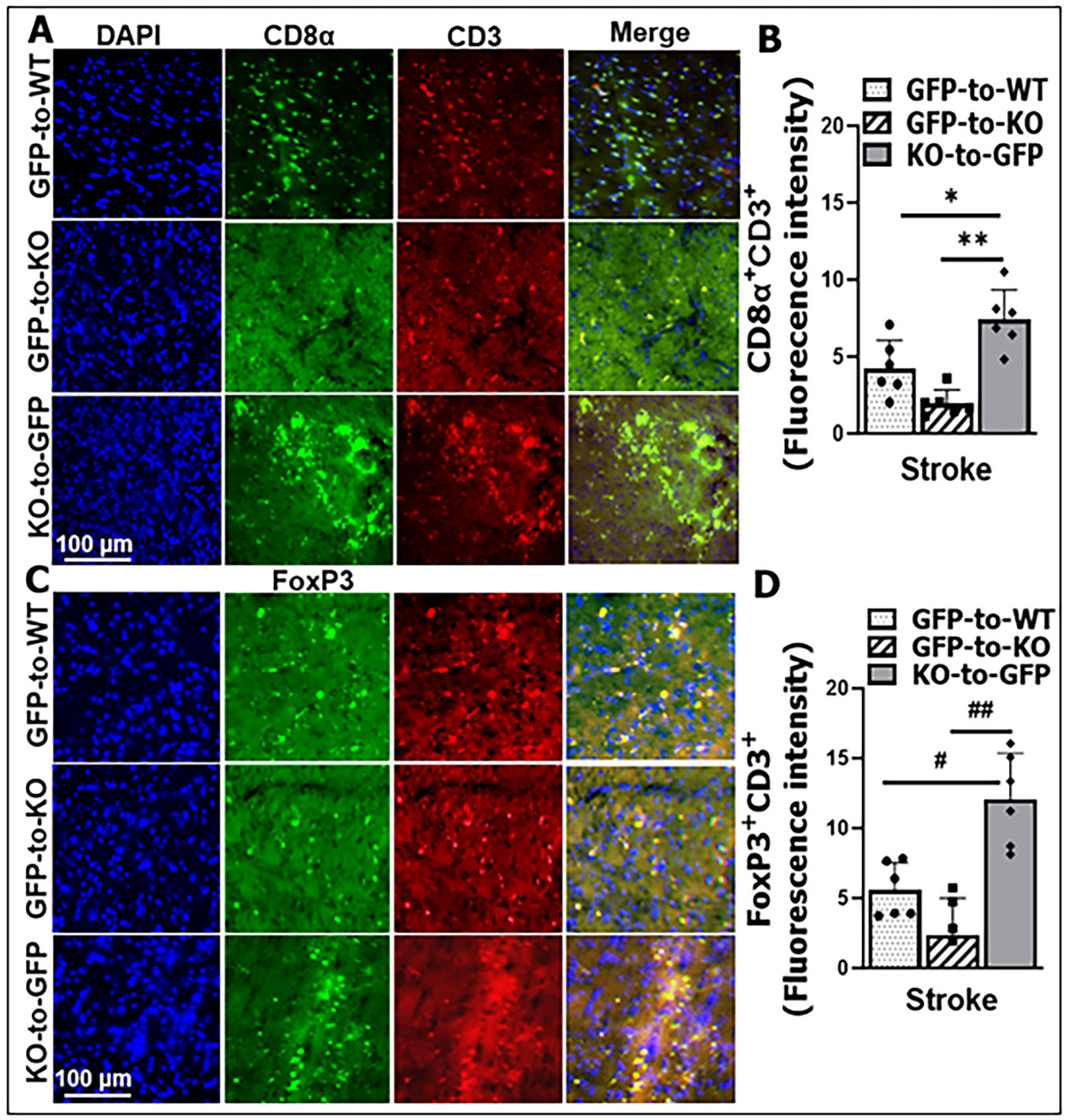
Post-stroke accumulation of CD8α^+^CD3^+^ and FoxP3^+^CD3^+^ T cell subsets in BMCs at 3 days after stroke. A, C, representative GFP-to-WT (upper rows), GFP-to-KO (middle rows), and KO-to-GFP (bottom rows) fluorescence staining slices. B, quantification of florescence intensity of CD8α^+^CD3^+^ T cells. D, quantification of florescence intensity of FoxP3^+^CD3^+^ T cells. Ordinary one-way ANOVA (CD8α: F (2, 15) = 17.65, p = 0.0001; FoxP3: F (2, 15) = 20.55, p < 0.0001).

Cluster of Differentiation 3 (CD3) is expressed on all mature T cells (Bardal, Waechter, and Martin, 2011). To characterize the T-cell subsets infiltrating the brain in our BMC mouse models three days after stroke, we stained brain sections with anti-CD3, anti-CD8α, and anti-FoxP3 antibodies (Fig. 6). KO-to-GFP chimeras exhibited significantly higher CD8α^+^CD3^+^ (Fig. 6A, B) and FoxP3^+^CD3^+^ (Fig. 6C, D) signal intensities compared with GFP-to-KO and GFP-to-WT chimeras. These findings indicate that both CD8α^+^CD3^+^ (CD8 cytotoxic) and FoxP3^+^CD3^+^ (Treg protective) T cells infiltrate the brain following stroke.

## 4. Discussion

The interaction between CD200 ligand and its receptor (CD200R) has been implicated in aging, neuroinflammatory and neurodegenerative diseases (Denieffe, Kelly, McDonald, Lyons, and Lynch, 2013; Ngwa and Liu, 2019; Pfeifer et al., 2023; Walker et al., 2009). However, the role of peripheral CD200R signaling in cerebral ischemia remains poorly understood. In this study, we generated BMC mouse models to test the hypothesis that peripheral CD200R signaling plays a critical role in regulating post-stroke inflammation in aged mice. Our BMC models achieved replicable and high immune cell reconstitution rate (>80 %) without developmental abnormalities or post-transplant mortality. Using these models, we identified several significant findings. Loss of peripheral CD200R signaling exacerbated post-stroke inflammation; while the central (brain) CD200R signaling had limited effects. Notably, compared to other peripheral immune cells, T cells more easily infiltrated into the ischemic brain when they lost CD200R. Corresponding with the aggravating inflammatory responses mounted in the brain, loss of CD200R in peripheral leukocytes induced worsened stroke outcomes, an effect not seen after CD200R was deleted in brain cells (R. M. Ritzel et al., 2019). To our knowledge, this is the first study to demonstrate distinct effects of peripheral versus central CD200R signaling in the context of stroke. Of note, our pilot studies have not found any sex differences in CD200 or CD200R expression in mice. However, to avoid any sex biased outcomes, we used chimeras of both sexes in this study, and no sex differences were found in immune response (Suppl, Fig. 3, for T cell subtypes) and stroke outcome data after sex stratified T tests.

The CD200R family comprises several isoforms; however, CD200R1 functions specifically as an inhibitory receptor involved in regulating immune response (Vaine and Soberman, 2014). Although other isoforms of the CD200 receptor (CD200RLs or CD200R2~4) also exist, it has been established that CD200 is not a ligand for these isoforms (Hatherley, Cherwinski, Moshref, and Barclay, 2005; Wright et al., 2003) and the function of these isoforms is uncertain (Gorczynski et al., 2004; Holmannová et al., 2012). An important advantage of using transgenic mice with a genetic deletion of CD200R, rather than CD200, is that any observed effect can be attributed specifically to the loss of CD200R-mediated inhibitory signaling, without confounding contributions from dual activating and inhibitory pathways involving CD200 (Gorczynski et al., 2004). As a result, genetic deletion of CD200R selectively removes the inhibitory pathway but leaves the activatory pathways intact (Ritzel et al., 2019).

The CD200-CD200R signaling axis has been implicated in experimental allergic encephalomyelitis (EAE) and various neurodegenerative diseases (González and Pacheco, 2014; Kotwica-Mojzych et al., 2021; Manich et al., 2019; Ngwa and Liu, 2019; Shen et al., 2024; Valente et al., 2017). The specific immune cell population on which CD200 exerts its inhibitory effect remains controversial. Previous studies have shown that CD200-CD200R interaction plays a regulatory role in modulating microglial activation under conditions of chronic and acute brain inflammation (Lyons et al., 2007). Disruption of this signaling pathway has also been shown to exacerbate microglial activation and accelerated dopaminergic neurodegeneration in a rat model of Parkinson’s disease (S. Zhang et al., 2011). However, our study using a stroke model (Ritzel et al., 2019), together with others, suggested that adult microglia express near null CD200R, whereas brain-infiltrating lymphocytes and myeloid cells express abundant CD200R (Pujol et al., 2024; Ritzel et al., 2019). These results indicate that CD200-CD200R signaling may primarily regulate peripheral immune cell activation rather than directly modulating microglial activity.

In the current study using BMC mouse models, we observed a significant increase specifically in T cell infiltration in KO-to-GFP compared with GFP-to-KO mice at 3 days post-stroke (Fig. 3B), indicating the removal of the "brake" imposed by CD200-CD200R interaction (Ngwa and Liu, 2019) has a significant role in facilitating T cell activation. The marked increase in T cells including CD8α^+^ and FoxP3^+^ (Treg) cells (Fig. 6) was accompanied by elevated levels of pro-inflammatory cytokines in brain lysates (Fig. 4), and correlated with worsened stroke outcomes (Fig. 5). This suggests T cell infiltration is sufficient to impact on stroke. Previous studies using bone marrow chimera mice have demonstrated that donor-derived T cells infiltrating into the brain after stroke, amplifying the neuroinflammatory response(Lee and McCullough, 2022; Lei et al., 2021; Wang et al., 2022). T cell infiltration has been detected as early as 24 h post-MCAO in animal models, with peak infiltration occurring between 3 and 5 days in transient MCAO models (Li et al., 2023; Planas, 2018; Tang, Zheng, and Yenari, 2012; Živančević, Lović, Andjus, and Radenović, 2021). This reflects a broad immune response to stroke, involving both the innate and adaptive immune systems. T cells infiltrating into the ischemic brain are notorious for their capacity to release a vast amount of inflammatory cytokines (Chamorro et al., 2012; Deng, Carter, Traystman, Wagner, and Herson, 2014; Heindl et al., 2021; Zhang et al., 2021) that can cause secondary neuronal death. Our data showed that CD200R deletion in T cells is sufficient to significantly exacerbate stroke outcomes, suggesting 1) CD200R is abundantly expressed on T cells; and 2) other immune cells may be subjected to multiple intrinsic inhibitory signals so that CD200R alone is insignificant in controlling activation of these cells. It is not surprising that CD200R deletion in peripheral leukocytes (KO-to-GFP) leads to increased infiltration of both “beneficial” (FOXP3^+^) and “detrimental” (CD8^+^) T cells compared to other chimeras, but our data pointed to an overall detrimental effects of T cells at the sub-acute time point of stroke.

Our group, along with others, have published accumulating data to highlight the use of BMC to investigate the differential impact of central versus peripheral immune responses in stroke (Duan, Xu, Li, Feng, and Chen, 2024; Ngwa et al., 2024; Ritzel et al., 2019; Tang et al., 2012). In our previous studies, mice were anesthetized using ketamine-xylazine, and the head was shielded with a lead screen during irradiation to minimize radiation-induced disruption to the blood-brain barrier (BBB) and the brain’s microenvironment, while allowing for peripheral immune system reconstitution with donor BM cells (Krishnan et al., 2021; Ngwa et al., 2024). However, this method has been controversial as the skull was spared from irradiation and its bone marrow could contaminate the reconstitution (Cugurra et al., 2021; Krishnan et al., 2021). In the current study, we implemented whole-body irradiation without head shielding (non-head-shielded chimeras) to achieve all bone marrow reconstitution including that of the skull. Specifically, we reduced X-ray exposure to two doses of 250 cGy instead of the 500 cGy initially used (Ngwa et al., 2024) to mitigate the irradiation induced inflammatory responses. We believe this method (no brain shielding and reduced X-ray exposure x 2 doses) has made our findings more valid and convincing.

There are limitations in our study that must be noted when interpreting the data. Due to the complexity of generating bone marrow chimeras and the increased frailty of aged mice, we restricted our analyses to the sub-acute post-stroke phase (3 days post-MCAO) to avoid high mortality in these aged chimeras after stroke. Hence the behavior tests in the current study were limited to sensorimotor tests and cognitive tests were not feasible at the sub-acute timepoint. Although the infarction is already mature (Fig. 5) (Al Mamun et al., 2020; Liu et al., 2009) and peripheral immune cell infiltration peaks by this time (Garcia-Bonilla et al., 2016), the effects of central vs. peripheral CD200R on stroke at chronic phase (e.g., 14–28 days) remain unclear. Future studies are warranted to address the long-term effects and cellular dynamics during this later phase. Another caveat is that we did not examine the peripheral organ dysbiosis (e.g., intestine) as the BM reconstitution may affect the gut microbiome and in turn affect the gut-brain inflammatory axis (Katiraei et al., 2022; Khalil and Maher, 2024; Korf, Ganesh, and McCullough, 2022). Our ongoing studies are investigating the impact of peripheral CD200-CD200R signaling on gut dysbiosis after stroke; one chronic timepoint (30d after MCAO) will be included and cognitive behavior tests will be examined.

In conclusion, our findings highlight a critical role for peripheral CD200R signaling in regulating post-stroke inflammation compared to the central CD200R. Loss of peripheral CD200R led to greater infiltration of T cells into the ischemic brain, and worsened stroke outcomes. These results suggest that CD200R expression on T cells, rather than central microglia, plays a key regulatory role in controlling neuroinflammation and recovery after stroke in aged mice.

## Supplementary Material

Supplementary Figures

## Appendix A. Supporting information

Supplementary data associated with this article can be found in the online version at doi:10.1016/j.brainresbull.2025.111686.
