# Supplementary material for: Peripheral CD200R signaling: A critical regulator of post-stroke inflammation in aged mice
Source: Brain Res Bull. Author manuscript; Available in PMC 2026 Mar 31. (PMC13036624; doi:10.1016/j.brainresbull.2025.111686)
Supplement: Supplementary Figures [file NIHMS2153339-supplement-Supplementary_Figures.pdf]

Supplementary figures

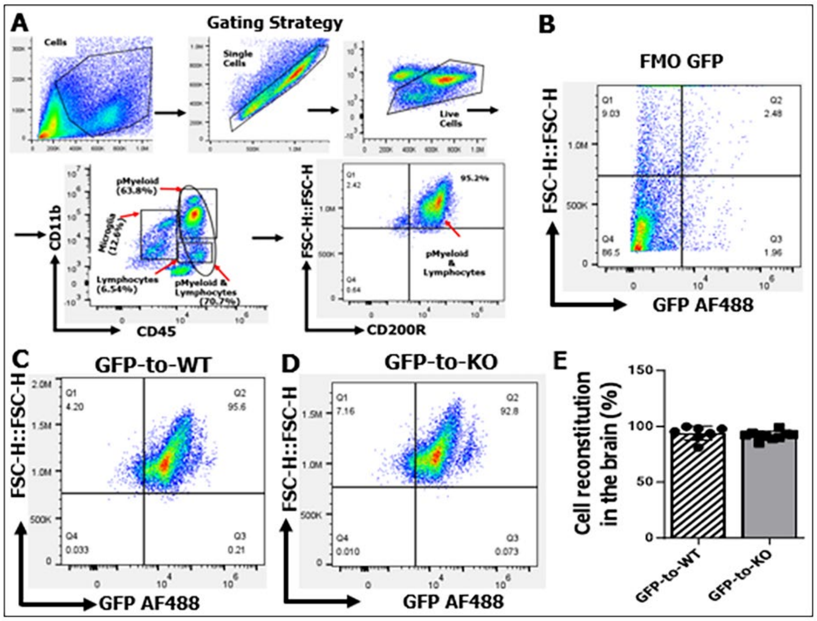

Suppl 1. Validation of bone marrow chimerism (Immune cell infiltration) in brain tissue after 3-days of stroke. **A**, gating strategy. **B,** Fluorescence minus one (FMO) for mouse lacking GFP cells in the brain. **C,** (GFP as donor, WT as recipient), **D** (GFP as donor, KO as recipient). **E** quantification of GFP^{+} cell reconstitution in brain, showing > 80% of GFP^{+} leucocytes in both WT and KO recipients. GFP-to-WT = peripheral and central CD200R intact; GFP-to-KO, peripheral CD200R intact and central CD200R absent. FMO, fluorescence-minus-one.

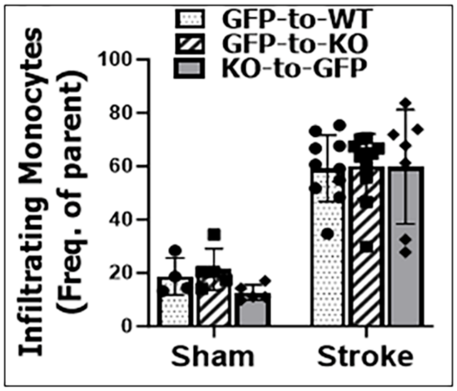

Suppl 2. pMyeloid infiltration in ischemic brains of chimeras after 3-days of MCAO. Quantification of monocyte in sham and ischemic mice. n = 4-6 for sham and 10-14 for the ischemic mice per group. Two-way ANOVA with Tukey's multiple comparisons test. GFP-to-WT = peripheral and central CD200R intact; GFP-to-KO, peripheral CD200R intact and central CD200R absent; KO-to-GFP, peripheral CD200R absent and central CD200R intact.

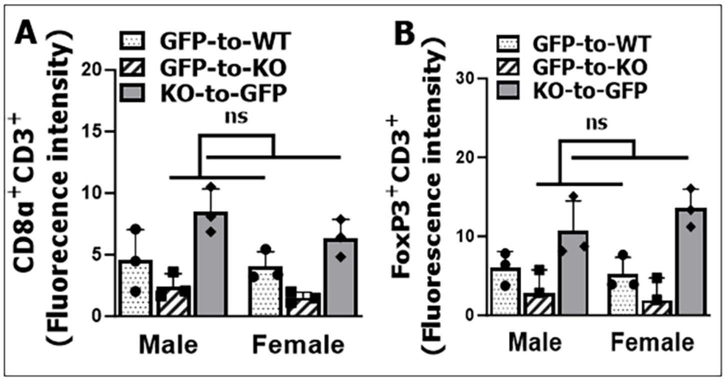

Suppl 3. Post-stroke accumulation of $CD8\alpha^{+}CD3^{+}$ and $FoxP3^{+}CD3^{+}$ T cell subsets in ischemic brains of chimeras after 3-days of MCAO in males and females. Quantification of fluorescence intensity. n = 3 for male and female per group. 2way ANOVA with Tukey's multiple comparisons test.
